# Transcriptional Regulation of Early T-Lymphocyte Development in Thymus

**DOI:** 10.3389/fimmu.2022.884569

**Published:** 2022-03-31

**Authors:** Xueyang Bao, Yingyu Qin, Linrong Lu, Mingzhu Zheng

**Affiliations:** ^1^Department of Pathogenic Biology and Immunology, Jiangsu Provincial Key Laboratory of Critical Care Medicine, School of Medicine, Southeast University, Nanjing, China; ^2^Shanghai Immune Therapy Institute, Renji Hospital, Jiao Tong University School of Medicine, Shanghai, China; ^3^Institute of Immunology, School of Medicine, Zhejiang University, Hangzhou, China

**Keywords:** transcriptional regulators, T-lymphocytes, double negative (DN) cells, double positive (DP) cells, single positive (SP) cells, T cell receptor (TCR)

## Abstract

T-lymphocytes play crucial roles for maintaining immune homeostasis by fighting against various pathogenic microorganisms and establishing self-antigen tolerance. They will go through several stages and checkpoints in the thymus from progenitors to mature T cells, from CD4^-^CD8^-^ double negative (DN) cells to CD4^+^CD8^+^ double positive (DP) cells, finally become CD4^+^ or CD8^+^ single positive (SP) cells. The mature SP cells then emigrate out of the thymus and further differentiate into distinct subsets under different environment signals to perform specific functions. Each step is regulated by various transcriptional regulators downstream of T cell receptors (TCRs) that have been extensively studied both *in vivo* and *vitro via* multiple mouse models and advanced techniques, such as single cell RNA sequencing (scRNA-seq) and Chromatin Immunoprecipitation sequencing (ChIP-seq). This review will summarize the transcriptional regulators participating in the early stage of T cell development reported in the past decade, trying to figure out cascade networks in each process and provide possible research directions in the future.

## Introduction

T cells widely participate in the innate and adaptive immune responses throughout the lifetime. T cell development is tightly regulated by numerous factors including transcriptional and epigenetic regulators. The proper development and differentiation of thymocytes is the foundation for the function of the immune system.

There is no doubt that the thymus is the fundamental place of thymocytes development that is highly organized, where thymocytes go through several stages and checkpoints before maturation and under-control of a network of multiple players (1, 2). Thymocyte development is driven by TCR activation and can be disrupted by defects in signaling components involved in the TCR signaling pathways (3, 4).

The early thymic precursor (ETPs) that come from bone marrow will go through different thymocyte developmental stages including CD4^-^CD8^-^ double negative (DN), CD4^+^CD8^+^ double positive (DP) and CD4^+^CD8^-^ or CD4^-^CD8^+^ single positive (SP). Then, mature SP cells will migrate to the periphery. Particularly, the DN population can be divided into four stages according to the expression of CD25 and CD44, starting from DN1 (CD44^+^CD25^-^), followed by DN2 (CD44^+^CD25^+^), DN3 (CD44^-^CD25^+^) and DN4 (CD44^-^CD25^-^) (5). In addition, the DN1 cells are known as ETPs. There are several check points during T cell development. β-selection is the first major checkpoint occurs at the DN3 stage. At this stage, a properly rearranged TCRβ chain will be produced that mediated by recombinant activating genes 1 and 2 (RAG1 and RAG2). Cells with successful β-selection downregulate the expression of CD25 and become DN4 cells, which then progress to the DP cells through the immature CD8 single positive (ISP) stage. In contrast, unsuccessful β-selection of DN3 cells will undergo apoptosis.

At the DP stage, TCRα gene rearrangements initiate and mature αβ-TCR will be produced. Subsequently, thymocytes must pass through both positive and negative selections to become mature T cells. Thymocytes with functional TCRs interact with the major histocompatibility complex (MHC) on cortical epithelial cells (cTECs) presenting foreign antigens will survive (6). Thus, positive selection is vital for MHC restrictions. During negative selection, thymocytes respond to self-antigens presented by mTECs (medullary epithelial cells) will be eliminated. Finally, the selected thymocytes differentiate into mature SP cells, emigrate out of thymus to periphery, and then differentiate into distinct functional subsets such as regulatory T cells (Treg), helper T cells (Th) and cytotoxic T cells. Less than 5% DP thymocytes will survive during all the checkpoints.

Each developmental step requires the participants of transcriptional regulators that have been elucidated through advanced genomic techniques to identify the binding sites (7–9). The transcription factors bind to regulatory elements of target genes, such as promoters, enhancers or silencers, to regulate the gene expression. In this review, we will briefly summarize the critical transcriptional factors and related epigenetic regulators during the T-lymphocyte development reported in the past decade and provide a comprehensive understanding of the thymocytes regulation.

## DN Stages

Notch signaling is one of the most important pathways to initiate the transcriptional program of the progenitor cells (10). Firstly, Notch signaling induces T cell-specific transcription factor TCF-1 (T cell factor 1, encoded by *Tcf7* gene) expression at the ETP stage. Then lead to the activation of two major target genes, Gata3 and Bcl11b (B-cell lymphoma/leukemia 11B) (11). Three waves of chromatin remodeling were observed at the ETP, DN2b and SP stage respectively. TCF-1 is enriched at recognition sites and regulatory regions that become accessible during the ETP and DN2b wave and persist until maturation in both humans and mice (12, 13). TCF-1 deficiency at distinct phases redirects bifurcation among divergent cell fates and subdivide the DN cells to different clusters *via* scRNA-seq. In addition, TCF-1 directly binds and mediates chromatin accessibility contributing to tumorigenesis (14). Moreover, TCF-1 is also found to directly interact with actin-nucleating factor WASp by ChIP-seq to promote T cell development (15). Most recently, Notch1 target genes HES1 and HES4 have been reported to be upregulated in a Notch-dependent manner promoting early T-cell development (16). Collectively, these studies emphasize the essential role of TCF-1 and Notch signaling in regulating T cell development.

Gata3 and Bcl11b are the major targets of TCF-1. Gata3 is a member of the Gata transcription factor family, plays multiple roles in the transcriptional network of thymocyte development. Gata3 deficiency will affect T-cell survival, growth, commitment and progression into mature cells. Gata3 has been proved to be additionally required at the earliest stage of thymopoiesis for the development of ETP population and DN2 to DN4 stages, since the mRNA levels of Gata3 are gradually increased between the ETP and DN3 stages and slightly diminish again in DN4 cells (7, 17). In mouse DN4 cells, Gata3 is bound by F-box protein Fbw7 and augmented in Fbw7-deficient thymocytes (18), while it is negatively regulated by E-box binding protein HEB *via* Notch1 (19). Furthermore, Gata3 positively regulates Bcl11b at the transition stage of T cell commitment. Over 10 years ago, the important roles of Bcl11b in the differentiation and survival of DN cells have been revealed (20–22). It is stimulated not only by Notch signaling but also by MAP kinase-and Gsk3-dependent signaling. The kinetic modifications of Bcl11b in DN cells are somewhat different from the patterns observed in DP cells, suggesting the essential function of Bcl11b in DN to DP transition (23). In addition, the expression of Bcl11b is impaired in CD147 deficient mice which results in failed T cell identity determination (24). More interestingly, the intraepithelial lymphocytes are decreased when Bcl11b is deficient (25). Cooperating with Bcl protein, transcription factor NFATc1 also plays a critical role in DN thymocytes survival and differentiation (26). It is activated by IL-7-Jak3 signals during the DN1 to DN3 stages (27, 28).

The function of each RUNT-related transcription factors (Runx) family member is still poorly understood based on current studies. Nevertheless, it is well known that Runx family members, including Runx1, Runx2 and Runx3 are another crucial transcription factors facilitating early T cell development. The activity of Runx1 has been highlighted in the relationship with other key transcription factors such as Bcl11b and Pu.1, which regulate the dynamic changes of transcriptional signatures before and after T cell commitment respectively. In addition, enforced expression of Runx2 affects β-selection resulting in an expansion of DN cells (29). The intronic silencer *(S4*) of *Cd4* gene cooperates with RUNX which is involved in T-helper inducing POZ-Kruppel factor (ThPOK) pathway (30). Herein, Runx family members are involved in various stages such as β-selection of double-negative thymocytes (22). The hypomorphic mutation of Runx component core-binding factor β (Cbfβ) results in a consecutive differentiation block within the DN population, as evidenced by a decrease of ETP followed by an inefficient ETP-to-DN2 transition as well as DN2-to-DN3 transition (22, 31).

## DN-to-DP Transition

T cells that have formed a functional pre-TCR complex, consisting of CD3, TCRβ, and pre-TCRα, can develop into DP cells. As a consequence, pre-TCR signaling is required for thymocyte development from DN to DP cells, following by dozens of transcriptional responses to pre-TCR signaling (32, 33). Moreover, pre-TCR signaling leads to increased expression of the transcriptional repressor Bcl6 which is required for differentiation to DP cells (34). Another member of the Bcl family is the antiapoptotic molecule Bcl2, whose down regulation induces enhanced apoptosis during the transition from the DN3 to the DN4 stage and contribute to DN4 cell number reduction. While the proliferation of ISP thymocytes is compensated, the number of ISP cells is normal eventually (35, 36). The successful assembly of pre-TCR promotes rapid self-renewal of DN3b cells and sequentially differentiate into cycling DN4, CD8 ISP and early DP (eDP) blast cells, then stop proliferating to become quiescent late DP (lDP) cells (37). TCR has multiple gene segments as alpha, beta, gamma and delta (*Tcra, Tcrb, Tcrg* and *Tcrd*). Murine *Tcra* and *Tcrd* are organized into a single genetic locus (*Tcra/Tcrd* locus) that undergoes V(D)J recombination in DN thymocytes to assemble the *Tcrd* gene and in DP thymocytes to assemble *Tcra* gene, to generate diverse TCR repertoires (38, 39). In addition, the formation of a functional VDJ join signals is required for robust proliferation of DN thymocytes and their differentiation into DP cells, whereas *Tcrb* recombination is suppressed by allelic exclusion (40).

Subsequently, pre-TCR complexes activate Notch1, whose activation is essential for generating the huge pool of DP thymocytes as physiological Notch1 signals are highest expressed in DN3 cells and decreased in DP cells. Thus, Notch1 signaling is crucial and transiently upregulated in DN-to-DP transition. There are two types of Notch1 related transcriptional regulators which are activators and repressors. Notch1 can be activated by Delta-like Notch ligands such as DL4, which is critically regulated by Lunatic Fringe (Lfng) (41, 42). Another activator is Zmiz1, which is a stage-specific cofactor of Notch1. Withdrawal of Zmiz1 at the later pre-T cell stage impairs the DN-to-DP transition by inhibiting proliferation. Furthermore, the Zmiz1-deficient DN-to-DP defect can be rescued by enforced activation of Notch1 or its target gene (43). However, DN4 and DP cells will be oncogenic when Notch1 is activated inappropriately (44). The repressors of Notch1 are vital for homeostasis. It is confirmed that Notch1 signaling can be attenuated by Bcl6 (34), NKAP (45) and Early growth response 2 (Egr-2) (46) in ISP thymocytes. Forced expression of these repressors may result in a severe reduction of DP cells in the thymus. Furthermore, downstream transcriptional factors of Notch1 also influence DN-to-DP transition. Induced TCF-1 form complex with β-catenin that will lead to transcriptional activation of cell-fate specific target genes in the transition and DP thymocytes survival *via* canonical Wnt pathway. On the contrary, TCF-1 interacts with co-repressors such as Groucho/Transducin-like enhancer (GRG/TLE) and turns off-target gene expression in the absence of Wnt signals. In the absence of TCF-1, ISP thymocyte development is blocked which contributes to DP thymocytes reduction (47).

As we mentioned in the previous section, Runx1 binds to the *CD4* silencer and represses transcription factors in immature DN thymocytes followed by CD8 expression to promote DN-to-DP transition, then down regulate in DP stage (48). The growth rate of DN4 cells and the transition of DN4 to the DP stage are impaired by overexpressed Runx1, resulting in the substantial reduction of DP thymocytes (49). Coincidentally, a sequence-specific transcription factor Ets1 specifically associates with Runx1 in DN and TCF-1 in DP cells by binding distal nucleosome-occupied and depleted regions respectively (50). Another critical transcription factors family is Ikaros which transiently increased from DN to DP developmental stage (51). Nevertheless, Ikaros maybe not a conventional activator or repressor according to defined sets of genes (52). As a tumor suppressor, Ikaros directly repress most Notch target genes through genome-wide analyses, such as ChIP-seq (53). Furthermore, a newly reported transcriptional regulator, Zinc finger protein Yin Yang 1 (YY1), is functional in DN thymocytes survival and apoptosis by suppressing the expression of p53, which can eliminate thymocytes that fail to pass β-selection. Early ablation of YY1 caused severely impaired development to DP cells due to increased apoptosis of DN thymocytes that prevented the expansion of post-β-selection thymocytes (54). Nevertheless, the comprehensive mechanism of YY1 in thymocyte development remains unclear though it is essential for iNKT cell development by ChIP-seq analysis (55).

## DP-to-SP Transition

### DP Survival

Appropriate TCR signaling is crucial for the survival of DP thymocytes and determines positive or negative selection (56). Without proper selective signaling, DP cells will be eliminated by apoptosis within 3~4 days during this pre-selection period.

RORγt is one of the most important survival transcription factors in pre-selective DP cells that activates the gene encoding the antiapoptotic protein Bcl-x_L_. It is well-known that the γc cytokine receptor subunit provides critical signals for T cell survival and differentiation. Recently, it is found that RORγt is abundant in immature DP thymocytes and act through Bcl-x_L_ to reduce the surface expression of γc. More importantly, Ligons et al. demonstrate that loss of RORγt in mouse DP thymocytes is associated with increased γc surface abundance and this phenomenon can be restored by forced expression of Bcl-x_L_ in RORγt-deficient thymocytes (57). Moreover, RORγt can be upregulated by TCF-1. Both TCF-1 and RORγt-knockout DP thymocytes undergo similarly accelerated apoptosis, while only in the presence of RORγt, the activation of TCF-1 by stabilized β-catenin can enhance DP thymocyte survival. Specifically, RORγt overexpression could rescue TCF-1 deficient DP thymocytes from apoptosis but overexpressed TCF-1 in RORγt^-/-^ DP thymocytes doesn’t show any rescue, which indicate that RORγt acts downstream of TCF-1. In addition, TCF-1 directly interacts with the promoter region of RORγt and induces its activity (58, 59). According to the most recent studies, TCF-1 may cooperate with transcription factors Zeb family member Zeb1 to participate in the cell cycle and TCR signaling by transcriptomic analysis (60).

Both Bim (Bcl2l11) and Nur77 are TCR-induced proteins with pro-apoptotic function. Bim is important for clonal deletion whereas Nur77 is often dispensable but able to influence late DP thymocytes apoptosis (61, 62).

Interestingly, nuclear speckle-related protein 70 (NSrp70) is selectively expressed on developing thymocytes and highest at DP stage. NSrp70 could regulate cell cycle and survival of thymocytes by governing the alternative processing of various RNA splicing factors, such as oncogenic serine/arginine-rich splicing factor SRSF1 (63). This finding may provide a new angle to dig up larger scale of transcription network in DP survival.

### Positive Selection

The DP thymocytes will undergo positive selection in the cortex of the thymus by recognizing antigen-MHC complex presented by cTECs and transducing intra-thymic TCR signals, then become CD4^+^ or CD8^+^ expressing SP cells. Calcium flux signaling is required for positive selection of T cells. Our results demonstrate a newly discovered adaptor named Tespa1 (Thymocyte-expressed, positive selection-associated 1) is essential for positive selection by modulating the interaction with a Ca^2+^ release channel - inositol 1,4,5-trisphosphate receptor (IP3R) which express on ER membranes (64–66).

The positive selection is also induced by forkhead box (Fox) family. In a way, the pioneer transcription factors Foxa1 and Foxa2 (forkhead box protein A) regulate alternative RNA splicing during thymocyte positive selection. Another Fox protein Foxo1 may induce the selection and maturation of DP thymocytes that can be accelerated in the deficiency of transcription repressor Gfi1(Growth factor independent 1). Thus, the Gfi1-Foxo1 axis shapes the proper generation of SP cells (67, 68). Additionally, Egr-2 also regulates the survival of stage-specific thymocytes and enhanced the maturation of DP cells into SP cells in thymus (46, 69).

Lastly, the achievement of positive selection is inseparable from epigenetic regulation which cross-work with transcriptional signals. HDAC7 (Histone deacetylase 7) has been reported exporting from the cell nucleus during positive selection in mouse thymocytes and modifying genes to mediate the coupling between TCR engagement and downstream events that determine cell survival including MAPK activity (70).

### Negative Selection

Negative selection is critical to delete highly self-reactive thymocytes to prevent autoimmunity. The thymocytes who pass the negative selection will become mature T cells with low self-reactivity and export to periphery immune organs.

The proceed of negative selection depends on functional mTECs, whose development is powerfully promoted by transcription factors Foxn1 (forkhead box family N1) and Aire (autoimmune regulator), which control the differentiation and maturation repectively (71). Conditional Foxn1 knockout results in defective negative selection contribute to less clonal deletion of autoreactive thymocytes (72), which possibly attribute to abnormal mTECs. Therefore, the Foxn1-TEC axis has been considered to repair negative selection and rejuvenation of thymic involution which is critical for counteracting inflammaging (73). Foxn1 is also the downstream target of Wnts which are a large family to secret glycoproteins and participate in cell fate determination, migration, proliferation, polarity and death in TECs. Existing evidences show Wnt4 and Wnt5b regulate Foxn1 expression in TECs through TCF-4 and LEF-1 by both autocrine and paracrine manners (74).

On the other hand, the function of mTECs is highly dependent on their characteristic features such as ectopic expression of tissue-restricted antigens (TRAs) and their master regulator Aire, whose expression is restricted to a mature subset of mTECs. Aire induces tissue-specific antigens to ensure negative selection by directly binding the promoter of the target gene within the medulla (75, 76). The transcriptional function of Aire in the process of mTECs adhesion is reconfirmed by CRISPR/Cas9 technology (77). Subsequently, it is shown that Aire targets 5’-URR (5’-untranslated regulatory region) of immune checkpoint HLA-G lead to increased intracellular HLA-G protein expression in TECs (78). Surprisingly, Aire can bind to sequence-independent epigenetic tags, such as unmethylated histone 3, and be recruited to a locus. After demethylation and Aire binding, Aire either directly enhances transcription or recruits other transcriptional activators (75).

In addition to the promoters, the transcriptional repressors of negative selection are indispensable. NCoR1 is a nuclear receptor co-repressor to connect repressive chromatin-modifying enzymes to gene-specific transcription factors. NCoR1 restrains negative selection by repressing pro-apoptotic factor Bim expression, which is expressed elevated in the absence of NCoR1. NCoR1-null thymocytes show excessive negative selection and reduced mature SP thymocytes (79–81). NCoR1 interacts with a predominant member of the HDAC family named HDAC3 which is a major and specific molecular switch that is crucial for mTECs differentiation and highly specific to histone deacetylases (82). Capicua (CIC) (83) and Sphingomyelin microdomains (SM) (84) also work as repressive factors together to ensure the proper negative selection and prevent autoimmunity.

## Discussion

**Figure 1:**
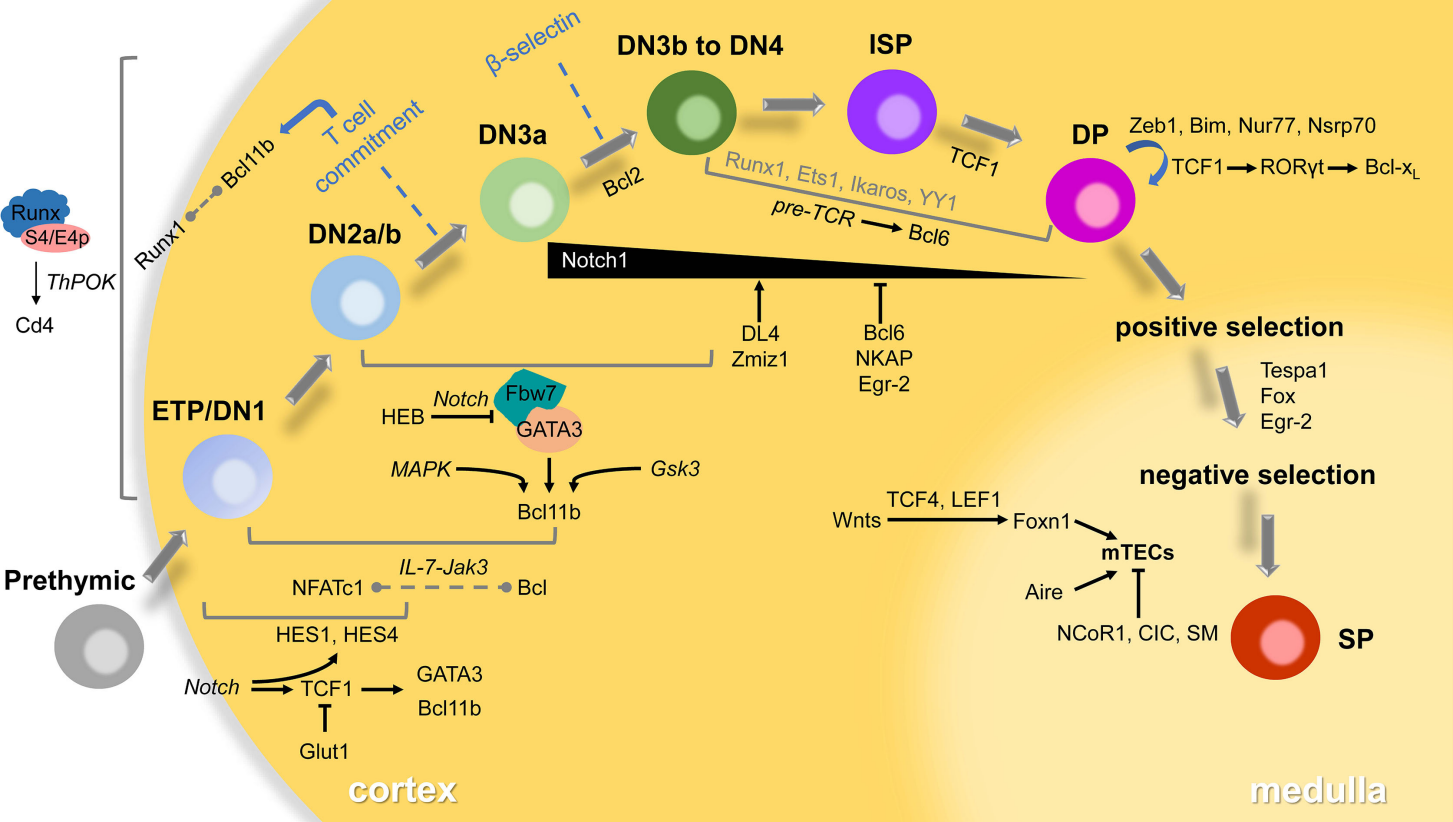
Transcriptional regulators in thymocyte development. T-lymphocytes go through several distinct stages according to expressed TCRs or surface markers in the thymus. The developing cells will pass checkpoints (β-selection, positive selection and negative selection) to become mature SP thymocytes, then emigrate to the periphery to differentiate to functional subsets. Most of the transcriptional regulators play roles in specific stages *via* small networks involving signaling in italics. Some interaction between factors remains unclear that displayed in the dotted line. The participants of a few transcriptional factors in grey are also poorly understood.

We conclude the map of T-lymphocyte development in the thymus and related transcriptional regulators that have been reported in the past decade (**Figure 1**), hoping to give some clues or inspiration to the future research. These selected regulators may have redundant or opposite functions in the thymocyte’s maintenance, proliferation, differentiation and maturation. Indeed, our understanding of the early stage of T lymphocytes development is still limited yet, the modulators we reviewed here are still poorly understood. Surprisingly, in recent years, more and more advanced techniques including various sequencing are invented or improved in order to elucidate the function of transcription factors involved in T cell development. However, the regulatory network among them and the precise mechanism still need further investigation both *in vivo* and *vitro* using ingenious animal models and molecular biological approaches.

## Author Contributions

XB and YQ prepared the initial draft. LL and MZ revised and finalized the manuscript. All authors contributed to the article and approved the submitted version.

## Funding

This work was supported by the National Natural Sciences Foundation of China (82171717 to MZ and 32100712 to YQ).

## Conflict of Interest

The authors declare that the research was conducted in the absence of any commercial or financial relationships that could be construed as a potential conflict of interest.

## Publisher’s Note

All claims expressed in this article are solely those of the authors and do not necessarily represent those of their affiliated organizations, or those of the publisher, the editors and the reviewers. Any product that may be evaluated in this article, or claim that may be made by its manufacturer, is not guaranteed or endorsed by the publisher.
